# Characterization of the complete mitochondrial genome of *Hyphessobrycon herbertaxelrodi* (Characiformes, Characidae) and phylogenetic studies of Characiformes

**DOI:** 10.1080/23802359.2020.1831986

**Published:** 2020-10-27

**Authors:** Kun Zhang, Pinglin Cao, Xiaolong Yin, Jian Chen, Pengxiang Yuan, Zengliang Miao, Hongling Ping, Hua Zhang, Bingjian Liu, Yuanpei Gao

**Affiliations:** a Marine Science and Technology College, Zhejiang Ocean University, Zhoushan, China; b Zhoushan Fisheries Research Institute of Zhejiang Province, Zhoushan, China; c Department of Food Science and Pharmacy, Zhejiang Ocean University, Zhoushan, China; d Key Laboratory of Tropical Marine Bio-resources and Ecology, Chinese Academy of Sciences, Guangzhou, China

**Keywords:** *Hyphessobrycon herbertaxelrodi*, mitochondrial genome, phylogenetic analysis, Characiformes

## Abstract

In this study, the complete mitochondrial genome of *Hyphessobrycon herbertaxelrodi* is presented, and we also discussed its mitochondrial characteristics. The full length of the mitochondrial genome was 17,417 bp, including 13 protein coding genes (PCGs), 2 ribosomal RNAs (12S and 16S), 22 transfer RNA genes, 1 non-coding control region (D-loop), and 1 origin of replication on the light-strand. The total nucleotide composition of mitochondrial DNA was 29.76%A, 29.88%T, 25.35%C, 15.01%G, and AT was 59.64%. The phylogenetic tree suggested that *H. herbertaxelrodi* shared the most recent common ancestor with *Astyanax giton*, *Grundulus bogotensis*, *Astyanax paranae,* and *Oligosarcus argenteus*.

Hyphessobrycon is one of the largest genera in the family Characidae comprising about 130 species distributed from Southern Mexico to the Río de la Plata in Argentina (Carvalho and Francisco 2013; Ingenito et al. 2013). The black neon tetra (*H. herbertaxelrodi*) is a well-known freshwater species (Gimeno et al. 2016). It is an ornamental fish, and acidic soft water will make the fish show its color. *Hyphessobrycon herbertaxelrodi* inhabits streams and lakes, likes to swim in groups, has a mild temperament, is omnivorous, and feeds on worms, crustaceans and plants. In this study, we described the complete mitochondrial genome of *H. herbertaxelrodi* and explored the phylogenetic relationship within Characiformes, to gain its molecular information and thus contribute to facilitate future studies on population genetic structure and phylogenetic relationships.

The sample of *H. herbertaxelrodi* used in this research was from Zhejiang Oceanography Laboratory (No. 20190825hld35). Total genomic DNA was extracted from a single specimen (SHOUBC72_1) using the improved method with multi-well plates (Pall Corporation) (Yue and Orban 2005). Subsequently, based on the existing complete mitochondrial gene of *Salminus brasiliensis* (KM245047.1) (Brandão-Dias et al. 2016), 18 pairs of primers were designed, the samples were amplified by PCR, and then sequenced using Sanger sequencing technology. The complete mitochondrial genome was annotated using Sequin version 15.10 (http://www.ncbi.nlm.nih.gov/Sequin) and tRNAscan-SE version 2.0 (http://trna.ucsc.edu/tRNAscan-SE/) (Patricia and Todd 2019). The whole mitochondrial genome of *H. herbertaxelrodi* was a closed circular molecule composed of 17,147 bp (GenBank accession no. MT769327.1), which was very similar to other typical vertebrate mitochondria (Boore 1999; Zhu et al. 2018). The complete mitochondrial genome contains 13 protein-coding genes (PCGs), two ribosomal RNA genes (12S rRNA and 16S rRNA), 22 transfer RNAs (tRNA) genes, and a putative control region (D-Loop) and one origin of replication on the light-strand (OL). The overall base composition is 29.76%A, 29.88%T, 25.35%C, 15.01%G, respectively, with a slight AT bias (59.64%). Most genes of *H. herbertaxelrodi* mitochondria are encoded on the H-strand, and only ND6 and eight tRNA (Gln, Ala, Asn, Cys, Tyr, Ser, Glu, and Pro) genes are encoded on the L-strand. In the 13PCGs gene, except that the COI starts with GTG, the others use ATG as the start codon, which is very common in vertebrate mtDNA (Prabhu et al. 2020). Most of the termination codon TAA or T-, only the stop codon COI for AGG. The lengths of 12S rRNA located between tRNA^Phe^ and tRNA^Val^ and 16S rRNA located between tRNA^Val^ and tRNA^Leu^ were 949 bp and 1500 bp, respectively. All 22 tRNAs distributed on the H and L-strands were between 66 and 74 bp in length. 14 tRNA genes were encoded on the H and eight on the L-strands. Most of tRNAs could form a common cloverleaf secondary structure, except tRNA^Ser(AGC)^ gene without DHU stem (Steinberg et al. 1993; Patricia and Todd 2019).

The phylogenetic tree based on the maximum-likelihood (ML) method was constructed to provide relationship within Characiformes. According to the Akaike Information Criteria (AIC), the most suitable nucleotide sequence model was selected through MrModeltest 2.3, and finally the most suitable model was GTR + I+G. The ML phylogenetic tree based on 13 PCGs of 42 species using the software PhyML 3.0 (Guindon et al. 2010). The phylogenetic tree suggests that *H. herbertaxelrodi* shares the most recent common ancestor with *Astyanax giton*, *Grundulus bogotensis*, *Astyanax paranae* and *Oligosarcus argenteus* (Figure 1).

**Figure 1.**
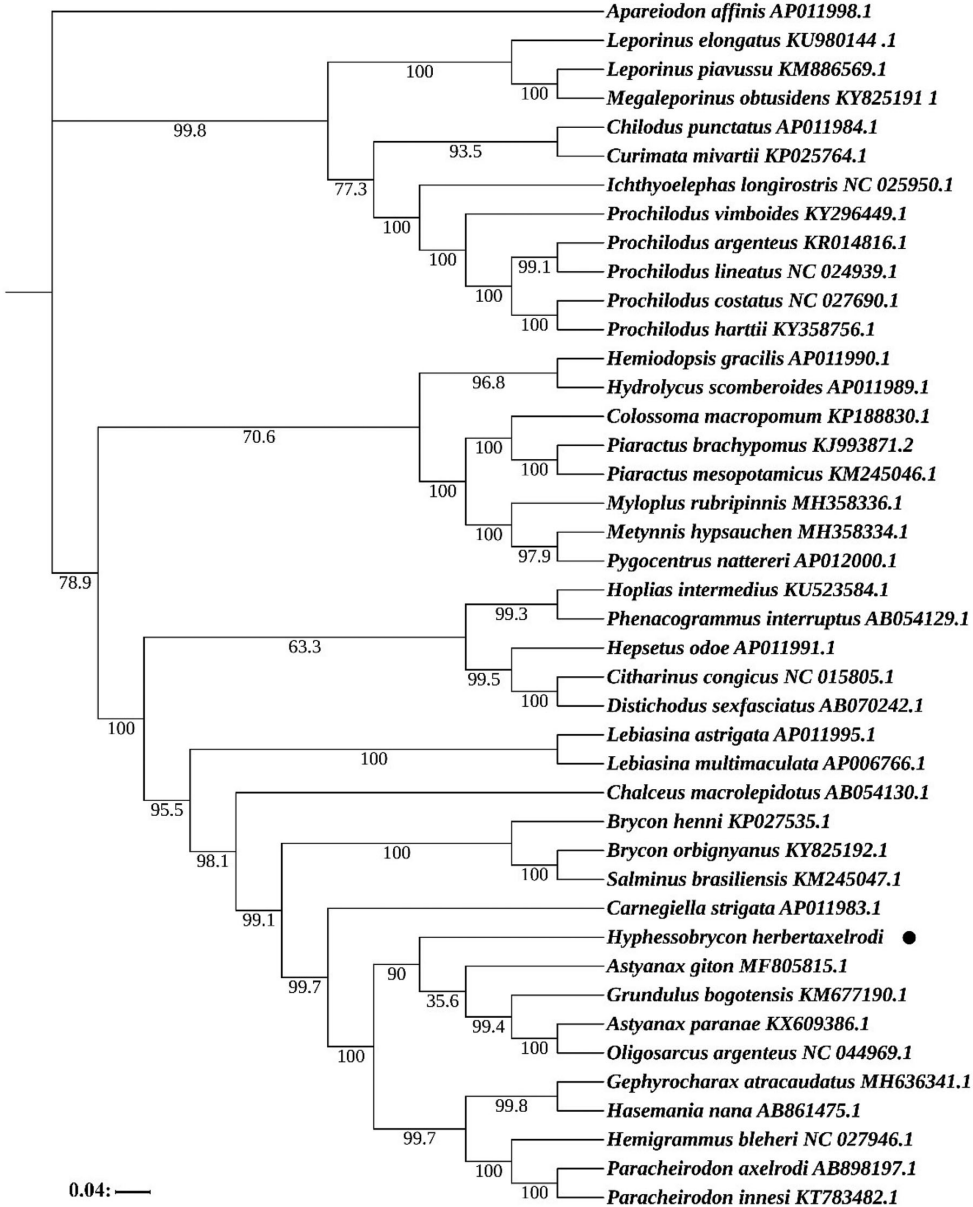
Maximum-likelihood (ML) phylogenetic tree based on 13 PCGs was used to study 42 species. The bootstrap values are based on 1000 resamplings. The number at each node was the bootstrap probability. The number before the species name is the GenBank accession number. The genome sequence in this study is labeled with a black dot.

## Data Availability

The data that support the findings of this study are openly available in “NCBI” at https://www.ncbi.nlm.nih.gov/, reference number MT769327.1.
